# A systematic review of metabolomic dysregulation in Chronic Fatigue Syndrome/Myalgic Encephalomyelitis/Systemic Exertion Intolerance Disease (CFS/ME/SEID)

**DOI:** 10.1186/s12967-020-02356-2

**Published:** 2020-05-13

**Authors:** Teilah Kathryn Huth, Natalie Eaton-Fitch, Donald Staines, Sonya Marshall-Gradisnik

**Affiliations:** 1 National Centre for Neuroimmunology and Emerging Diseases, Menzies Health Institute, Griffith University, Gold Coast, Australia; 2 School of Medicine, University of Notre Dame, Sydney, Australia

**Keywords:** Chronic Fatigue Syndrome/Myalgic Encephalomyelitis/Systemic Exertion Intolerance Disease (CFS/ME/SEID), Metabolomics, Metabolome

## Abstract

**Background:**

Chronic Fatigue Syndrome/Myalgic Encephalomyelitis/Systemic Exertion Intolerance Disease (CFS/ME/SEID) is a complex illness that has an unknown aetiology. It has been proposed that metabolomics may contribute to the illness pathogenesis of CFS/ME/SEID. In metabolomics, the systematic identification of measurable changes in small molecule metabolite products have been identified in cases of both monogenic and heterogenic diseases. Therefore, the aim of this systematic review was to evaluate if there is any evidence of metabolomics contributing to the pathogenesis of CFS/ME/SEID.

**Methods:**

PubMed, Scopus, EBSCOHost (Medline) and EMBASE were searched using medical subject headings terms for Chronic Fatigue Syndrome, metabolomics and metabolome to source papers published from 1994 to 2020. Inclusion and exclusion criteria were used to identify studies reporting on metabolites measured in blood and urine samples from CFS/ME/SEID patients compared with healthy controls. The Joanna Briggs Institute Checklist was used to complete a quality assessment for all the studies included in this review.

**Results:**

11 observational case control studies met the inclusion criteria for this review. The primary outcome of metabolite measurement in blood samples of CFS/ME/SEID patients was reported in ten studies. The secondary outcome of urine metabolites was measured in three of the included studies. No studies were excluded from this review based on a low-quality assessment score, however there was inconsistency in the scientific research design of the included studies. Metabolites associated with the amino acid pathway were the most commonly impaired with significant results in seven out of the 10 studies. However, no specific metabolite was consistently impaired across all of the studies. Urine metabolite results were also inconsistent.

**Conclusion:**

The findings of this systematic review reports that a lack of consistency with scientific research design provides little evidence for metabolomics to be clearly defined as a contributing factor to the pathogenesis of CFS/ME/SEID. Further research using the same CFS/ME/SEID diagnostic criteria, metabolite analysis method and control of the confounding factors that influence metabolite levels are required.

## Background

Chronic Fatigue Syndrome/Myalgic Encephalomyelitis/Systemic Exertion Intolerance Disease (CFS/ME/SEID) is a complex and chronic illness with an unknown pathophysiology [1, 2]. The global prevalence of CFS/ME/SEID ranges between 0.4 and 2.5% and it predominantly affects young adults between the ages of 20 to 40 years, with a higher proportion of females affected compared to males [3, 4]. CFS/ME/SEID is characterised by fatigue that is disabling, does not improve with rest and persists for at least 6 months [5]. In addition, CFS/ME/SEID patients also typically present with a consistent, yet diverse symptomatology including tender lymph nodes, low-grade fever, muscle pain, joint pain, recurrent sore throat, headaches, sleep disturbances, non-refreshing sleep, post-exertional malaise, changes in memory and concentration and gastrointestinal symptoms [5–7]. Patients often report a relapsing–remitting symptom pattern, with variations in the frequency, severity and duration [1, 8]. Consequently, many patients with CFS/ME/SEID experience significant decline in their physical, mental, social and occupational functioning, which reduces their personal activity levels and quality of life [9–11].

CFS/ME/SEID is a heterogenous condition of unknown aetiology and there is currently no diagnostic test. For the purpose of clinical and epidemiological research, case definitions are used to identify CFS/ME/SEID patients [5–7]. Diagnosis is made in accordance with specific clinical criteria including symptoms, illness onset and duration of fatigue suffered. A number of case definitions including the 1994 Fukuda, International Consensus Criteria (ICC), Canadian Consensus Criteria (CCC), Oxford and Institute of Medicine (IOM) [5–7, 12] have been developed to diagnose CFS/ME/SEID patients. Compared to the 1994 Fukuda criteria, the CCC requires the addition of neuroimmune exhaustion and autonomic and endocrine dysfunction, whilst the ICC includes temperature intolerances and the removal of the six months wait period. Exclusion criteria are similar across the case definitions where patients who have other disease processes—including iron deficiency, hypo- or hyper-thyroidism, diabetes, cancer, psychosis, epilepsy, cardiac conditions, sleep disorders, neurological impairments, gastrointestinal disorders or immune deficits—that may explain some of the CFS/ME/SEID symptoms, are excluded.

Metabolomics refers to the process of identifying metabolite substrates and products found in biofluids, cells and tissues of living systems [13, 14]. Metabolites have been identified to contribute to normal physiological functioning by driving cellular energy production and storage, signal transduction and apoptosis [15]. It has been proposed that the metabolite profile reflects underlying genetic and physiological processes of the living system, acting as a chemical and biological fingerprint. Metabolomics also provides an insight into the influence of external stimuli such as environment, diet and microflora on the functioning of the living system [16].

In metabolomics, the systematic identification of measurable changes in small molecule metabolite products have been identified in cases of both monogenic and heterogenic diseases [14]. Altered metabolomics may be associated with disease pathogenesis not previously considered to be of metabolic origin, such as cancer, cognitive disorders and respiratory pathologies [13, 14]. An advantage of investigating metabolite changes is that in contrast to an analysis of the genome, metabolite profiles can exhibit tissue specificity and temporal dynamics [15]. In addition, metabolite profiling using mass spectrometry or nuclear magnetic resonance allows for a significantly larger number of metabolite substrates to be assessed when compared to standard laboratory techniques; a total of 4229 metabolites have been identified in human samples from laboratory investigations [16, 17]. Biological samples including blood or urine can be assessed for metabolites targeting specific biochemical pathways or the analysis can be untargeted, identifying as many metabolites as possible [18]. The ability to perform metabolite analyses provides a technology platform which may be beneficial for investigating if metabolomics contributes to the pathogenesis of CFS/ME/SEID. Previous studies have identified irregularities in metabolites associated with energy metabolism, lipid metabolism, nucleotides, peptides and cofactors and vitamins. The aim of this systematic review was to evaluate if there is any evidence of metabolomics contributing to the pathogenesis of CFS/ME/SEID.

## Methods

### Literature search

The study was conducted according to the Preferred Reporting Items for Systematic Reviews (PRIMSA) guidelines. Databases including PubMed, Scopus, EBSCOHost (Medline) and EMBASE were searched using medical subject headings (MeSH) terms for Chronic Fatigue Syndrome, metabolomics and metabolome. The search was conducted using the Boolean operator AND to combine all of the expression cases for CFS/ME/SEID (including abbreviations) in conjunction with metabolomics. The Boolean operator OR was used to combine all expression cases for metabolomics OR metabolome. The following full-text terms were searched: CFS/ME AND metabolomics OR metabolome. Proximity operators were not used in the search. To avoid author selection bias in this systematic review, two separate authors conducted literature searches using the same method on different occasions. The first author (TKH) completed the primary search on 23 December 2019 and a secondary search was completed by the second author (NEF) on 17 January 2020. A final search was completed on 2 February 2020.

### Screening, inclusion and exclusion criteria

This systematic review was conducted to evaluate primary research that compared metabolite levels in biological samples (blood and urine) from CFS/ME/SEID patients with healthy controls (HC). All of the results from the literature search were imported into EndNote for storage and screening. Duplicate copies of papers were removed, and the following criteria were used by the authors to screen titles and abstracts: (i) all primary research comparing metabolite levels in blood or urine samples between CFS/ME/SEID patients and HC; (ii) CFS/ME/SEID diagnosis according to Fukuda, CCC or ICC; (iii) use of appropriate statistics to compare CFS/ME/SEID patients and HC cohorts; (iv) studies published between 1994 and 2019 to exclude non-Fukuda definitions prior to 1994; (v) human studies in adults aged 19 years and above; (vi) journal articles published in English; and (vii) free and paid full text publications of original research to exclude publication bias.

Studies were excluded if: (i) only one of the three keywords were included in the title or abstract; (ii) CFS/ME/SEID patient group was compared with other patient groups (e.g. irritable bowel syndrome, fibromyalgia etc.) and not HC; and (iii) studies including interventional assessments for pharmacological therapies or exercise programs.

### Selection of studies and data extraction

After the titles and abstracts were screened, the full text of eligible papers were analysed. The reference lists of eligible papers were also screened for any additional research papers. For all studies meeting the inclusion criteria listed above, the following details were extracted: (i) author, (ii) year, (iii) country, (iv) study design, (v) CFS/ME/SEID diagnostic criteria, (vi) metabolite analysis details and (vii) sample size of CFS/ME/SEID and HC cohorts. Data with statistical results defined by a p-value were then extracted for the primary outcome of metabolites in serum or plasma samples from CFS/ME/SEID patients compared with HC. Data with statistical results defined by a p-value for the secondary outcome of metabolites in urine samples from CFS/ME/SEID patients compared to HC were then extracted.

### Quality assessment

The Joanna Briggs Institute (JBI) Critical Appraisal Checklist for Case Control Studies was used to evaluate the included studies for quality and bias [19]. JBI Checklist numbers four, five and nine were excluded from the quality assessment due to specificity for interventional studies. All included papers were assessed as either meeting each of the remaining seven checklist criteria or not. For each adherence to a checklist item, a score of one was assigned, which was then converted to a total percentage score for each study [20]. Two authors (TKH and NEF) independently completed the JBI Critical Appraisal Checklist for all of the studies included in this review.

## Results

**Fig. 1:**
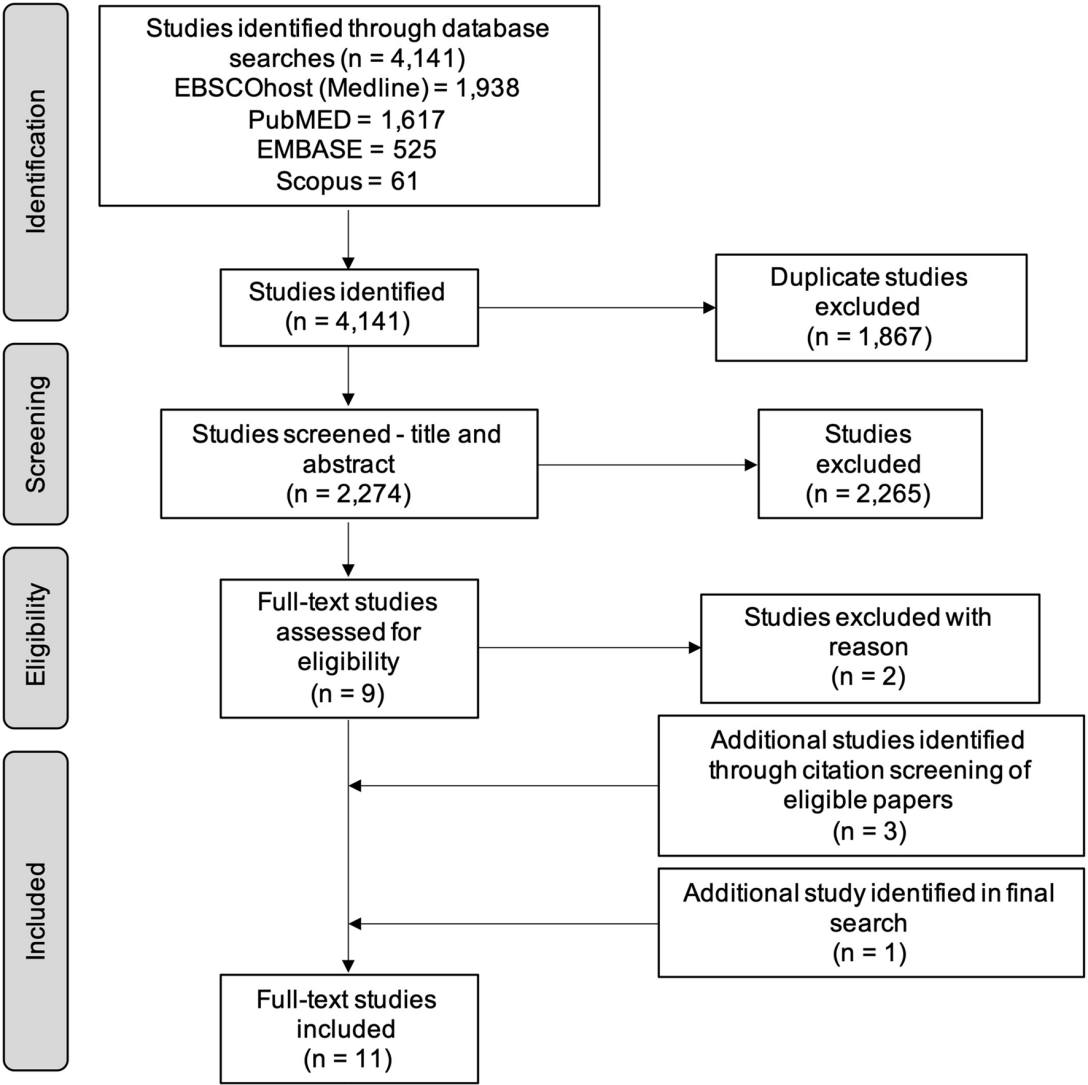
PRIMSA flow diagram of the literature search results for studies measuring metabolites in CFS/ME/SEID patients

A total of 4141 studies were identified from Medline (EBSCOhost) (1938), PubMED (1617), EMBASE (525) and Scopus (61). After the removal of duplicate studies, 2274 papers were screened according to the inclusion and exclusion criteria previously outlined. A PRIMSA flow diagram (Fig. 1) summarises the results for the literature search with the number of studies that were included and excluded.

### Overview of included papers

A total of 11 papers met the inclusion criteria for this literature search (Table 1). From the database searches, seven papers were identified and a further three studies were found in citation screening of the eligible papers. One additional paper was identified in a final search conducted on 2 February 2020 [21]. All of the results in this review were observational case control studies that compared metabolite levels in plasma, serum or urine samples in CFS/ME/SEID patients to HC. After separately completing the literature search and eligibility screening for this review, the authors reported no discrepancies in the results.

**Table 1:** Summary of studies meeting inclusion criteria

Author	Year	Country	Study design	CFS/ME/SEID diagnostic criteria	Metabolite			Sample size	
					Sample	Targeted/untargeted	Analysis method	CFS/ME/SEID	HC
Germain et al.	2020 [21]	USA	Case control	Fukuda	Plasma	Untargeted	Metabolon mass spectrometry	26 F	26F
Germain et al.	2018 [22]	USA	Case control	Fukuda	Plasma	Untargeted	Metabolon mass spectrometry	32 F	19 F
Nagy-Szakal et al.	2018 [29]	USA	Case control	Fukuda CCC	Plasma	Targeted and untargeted	Gas chromatography time-of-flight and liquid chromatography–tandem mass spectrometry	26	49
Germain et al.	2017 [23]	USA	Case control	Fukuda	Plasma	Untargeted	Q-exactive mass spectrometry	17 F	15 F
Yamano et al.	2016 [24]	Japan	Case control	Fukuda	Plasma	Untargeted	Capillary electrophoresis mass spectrometry	41 F 5 M	41 F 6 M
Fluge et al.	2016 [25]	Norway	Case control	CCC	Serum	Targeted	Gas chromatography–tandem mass spectrometry	162 F 38 M	67 F 35 M
Naviaux et al.	2016 [30]	USA	Case control	Fukuda CCC IOM	Plasma	Targeted	Triple quadrupole mass spectrometry	23 F 22 M	21 F 18 M
Armstrong et al.	2015 [26]	Australia	Case control	CCC	Serum Urine	Untargeted	Nuclear magnetic resonance spectrometry	34 F	25 F
Armstrong et al.	2012 [18]	Australia	Case control	CCC	Serum	Untargeted	Nuclear magnetic resonance spectrometry	6 F 5 M	5 F 5 M
Jones et al.	2005 [27]	United Kingdom	Case control	Fukuda Oxford	Plasma Urine	Targeted	Reversed phase chromatography	19 F 11 M	19 F 11 M
McGregor et al.	1996 [28]	Australia	Case control	Fukuda Oxford	Urine	Untargeted	Capillary gas chromatography–mass spectrometry	16 F 4 M	32 F 13 M

*F* female, *M* male

### Participant and study characteristics

Across the 11 studies, blood metabolites (in plasma or serum samples) were measured in a total of 467 CFS/ME/SEID patients and 362 HC (Table 1). Urine metabolites were measured in a total of 84 CFS/ME/SEID patients and 100 HC. A total of four different criteria were used to diagnose CFS/ME/SEID patients. In four studies, CFS/ME/SEID patients met the diagnostic criteria for 1994 Fukuda [21–24], three studies met the CCC [18, 25, 26], two studies met 1994 Fukuda and Oxford [27, 28], one study met 1994 Fukuda and CCC [29] and one study met 1994 Fukuda, CCC and IOM [30]. Four of the studies included only female participants [21–23, 26]. Seven studies measured metabolites in plasma samples and three studies measured metabolites in serum and urine samples using varying forms of extraction techniques and mass spectrometry. Two studies used the same Nuclear Magnetic Resonance Spectrometry analysis method and two studies used the same Metabolon Mass Spectrometry method [18, 21, 22, 26]. In seven of the studies, metabolite analysis were untargeted, three of the studies were targeted and one study performed both a targeted and untargeted assessment (Table 1).

### Blood metabolites—primary outcome

A total of 3394 blood metabolites (in plasma and serum samples) were assessed in 10 studies and 210 metabolites were found to be significantly different (decreased or increased) when CFS/ME/SEID patients were compared to HC (Table 2). One study presented the data split as males and females and as such, no combined data were available for this paper [30]. Given the large number of metabolites that were found to be significantly different between CFS/ME/SEID patients and HC in this systematic review, the major biochemical pathways associated with each significant metabolite are summarised in Table 3 [22]. Metabolites associated with the amino acid pathway were the most commonly impaired with significant results in seven out of the 10 studies.

**Table 2:** Study results for blood metabolite analyses

Author and year	Number of metabolites		Blood metabolite	
	Assessed	Significantly different in CFS/ME/SEID vs HC	Decreased in CFS/ME/SEID vs HC (p-value)	Increased in CFS/ME/SEID vs HC (p-value)
Germain et al. (2020) [21]	786	41	Cysteinylglycine (0.008) Hypotaurine (0.039) Indolelactate (0.006) Tryptophan betaine (0.035) Dehydroepiandrosterone sulfate (DHEA-S) (0.035) Epiandrosterone sulfate (0.035) Androstenediol (3alpha, 17alpha) monosulfate (2) (0.039) 5alpha-androstan-3beta,17alpha-diol disulfate (0.04) Androsterone sulfate (0.041) Androstenediol (3beta,17beta) disulfate (2) (0.044) Etiocholanolone glucuronide (0.049) Cortisone (0.004) Cortisol (0.02) Dihomo-linolenoyl-choline (0.019) Linoleoylcholine (0.036) Stearoylcholine (0.043) Docosahexaenoylcarnitine (C22:6) (0.025) Adrenoylcarnitine (C22:4) (0.029) Octanoylcarnitine (C8) (0.035) Decanoylcarnitine (C10) (0.049) Branched chain 14:0 dicarboxylic acid (0.049) 3-hydroxymyristate (0.013) 3-hydroxydecanoate (0.015) 3-hydroxylaurate (0.029) Cis-4-decenoate (10:1n6) (0.047) Valerate (5:0) (0.045) Phenylalanylalanine (0.007) Phenylalanylglycine (0.014) Valylleucine (0.046) Dimethyl sulfone (0.032) Piperine (0.027) Sulfate of piperine metabolite C16H19NO3 (3) (0.027) Stachydrine (0.044)	*N*-carbamoylalanine (0.049) 4-hydroxyglutamate (0.004) *N*-acetyl-1-methylhistidine (0.038) *N*,*N*,*N*-trimethyl-alanylproline betaine (TMAP) (0.039) Arachidoylcarnitine (0.025) Adipoylcarnitine (C6-DC) (0.042) Glycohyocholate (0.048) Gamma-glutamyltyrosine (0.045)
Germain et al. (2018) [22]	832	9	Gamma-CEHC (0.005) Alpha-CEHC glucuronide (0.018) Gamma-CEHC glucuronide (0.019) Inosine 5′-monphosphate (0.003) 2′-*O*-methylcytidine (0.009) Adenosine 3′-5′-cyclicmonophosphate (0.012)	Haem (0.002) Alpha-ketoglutarate (0.03) Gamma-glutamyl-threonine (0.003)
Nagy-Szakal et al. (2018) [29]	562	2	Carnitine-choline (0.017) Phosphatidylcholine (0.017)	None
Germain et al. (2017) [23]	361	65	Acetylcarnosine (0.0014) ATP (0.0024) ADP (0.0034) Glycochenodeoxycholate (0.0044) C3H4O2.3 (0.0050) 2-Methylglutaconic acid (0.0097) C20H34O4.4 (0.0032) Taurine (0.0073) 13-carboxy-alpha-tocopherol (0.0027) 4-Imidazolone-5-propanoate (0.0023) Sulfoglycolithocholate (0.0058) C4H6O3.3 (0.0074) Acetamidopropanal (0.0164) l-proline (0.0164) d-glucose (0.0091) l-erythrulose (0.0093) 2,3-epoxy-alpha-tocopherylquinone,5,6-epoxy-alpha-tocopherylquinone (0.0082) l-glutonate (0.0091) d-glutonate (0.0091) C20H32O4.21 (0.0034) Glycolothocholate (0.0075) CDP-Choline (0.0141) Glycocholate (0.0110) 25-hydroxyvitamin D3-26,23-lactone (0.0075) Lithocholate (0.0182) Glyoxylate (0.0073) Choline phosphate(1-)(0.0085) Succinylcarnitine (0.0271) 5-guanidino-2-oxopentanoic acid (0.0050) Phosphoanto-oxy-phisphonate-UDP-d-galactose (0.0110) *S*-(2-Methylpropanoyl)-dihydrolipoamide (0.0064) Ethanolamine phosphate (0.0201) *S*-[2-Carboxy-1-(1H-imidazol-4-yl)ethyl]-l-cysteine (0.0153) 3alpha,7alpha,12alpha,25-tetrahydroxy-5beta-cholestane-24-one/3alpha,7alpha,12alpha-trihydroxy-5beta-cholestanoate (0.0136) l-kynurenine/Fromyl-5-hydroxykynurenamine (0.0201) Fructoseglycine (0.0332) Oxaloacetate (0.0396) C27H46O4.3 (0.0259) 5-hydroxyindoleacatate (0.0328) 2-Keto-3-deoxy-d-glycero-d-galacto-nonic acid (0.0430) l-isoleucine, l-Leucine (0.0482) 13-carboxy-gamma-tocopherol (0.0475) d-Glucuronate 1-phosphate (0.0195) Nicotinamide (0.0396) 3-oxo-8(R)-hydroxy-hexadeca-6E10Z-dienoate_3-oxo-8(S)-hydroxy-hexadeca-6E10Z-dienoate (0.0264) Biotin (0.0363) 3-Hydroxyisovalerylcarnitine (0.0460) UTP (0.0461) 5-amino-1-(5-phospho-d-ribosyl)imidazole-4-carboxamide (0.0357)	Cis-11-Eicosenate (0.0069) C20H30O2.4 (0.0041) Cervonic acid C22:6(n-3), docosahexaenoate (0.0042) 3-(methylthio)propionate (0.0299) (Trans-vaccenate-elaidate-oleate (0.0375) 5,6-Dihydrothymine (0.0259) Urocanate (0.0392) N2-Formyl-N1-(5-phospho-d-ribosyl)glycinamide (0.0361) 4-Hydroperoxy-2-nonenal (0.0264) Glycero-3-phosphate (0.0333) Arachidonate, Eicosatetranoic acid (0.0240) Pristanic acid, pristanate (0.0290) Deoxyuridine (0.0296) Clupanodonic acid docosa-4,7,10,13,16-pentaenoic acid (0.0341) 4-Hydroxyphenylacatate/2-Hydroxyphenylacetate/3,4-Dihydroxyphenylacetaldehyde (0.0430) Spermidine (0.0464)
Yamano et al. (2016) [24]	144	6	Citrate (< 0.05) Isocitrate (< 0.05) Malate (< 0.05) Urea (< 0.01) Citrulline (< 0.01)	Ornithine (< 0.05)
Fluge et al. (2016) [25]	20	14	Lys (0.001) Leu (< 0.001) Phe (< 0.001) Tyr (< 0.001) Ile (< 0.001) Trp (0.009) Ala (0.027) Val (< 0.001) Met (0.017) Asx (< 0.001) His (< 0.001) Pro (< 0.001) Glx (0.029) SDMA (0.001)	None
Naviaux et al. (2016) [30]	612	61-Females	Ceramide(d18:1/25:0) (< 0.001) THC 18:1/24:0 (< 0.001) PC(16:0/16:0) (< 0.001) Lathosterol (< 0.001) PI(16:0/16:0) (< 0.001) Ceramide(d18:1/22:2) (< 0.001) Adenosine (< 0.001) Ceramide(d18:1/24:2) (< 0.001) THC 18:1/16:0 (< 0.001) 2-Octenoylcarnitine (< 0.001) GC(18:1/16:0) (< 0.001) Phenyllactic acid (< 0.001) Ceramide(d18:1/26:0) (< 0.001) Ceramide(d18:1/24:0) (< 0.001) DHC(18:1/16:0) (< 0.001) Ceramide(d18:1/26:2) (< 0.001) FAD (< 0.001) Ceramide(d18:1/16:0) (< 0.001) SM(d18:1/22:2) (< 0.001) Adenosine monophosphate (< 0.001) PI(38:3) (< 0.001) Chenodeoxycholic acid (0.007) Ceramide(d18:1/20:0) (0.007) Ceramide(d18:1/22:1 OH) (0.009) Ceramide(d18:1/18:2 OH) (0.009) Ceramide(d18:1/22:0) (0.010) Ceramide(d18:1/18:0) (0.010) Ceramide(d18:1/16:1 OH) (0.010) Ceramide(d18:1/24:2 OH) (0.012) SM(d18:1/16:0) (0.012) Ceramide(d18:1/24:1) (0.013) PI(34:1) (0.014) PI(36:0) (0.016) SM(d18:1/20:1) (0.016) 2-Arachidonylglycerol (0.018) THC 18:1/18:0 (0.018) Ceramide(d18:1/22:1) (0.019) PI(34:0) (0.020) Ceramide(d18:1/26:1 OH) (0.020) PI(38:4) (0.021) Hydroxyisocaproic acid (0.022) PC(30:0) (0.025) Cobalamin (0.026) Ceramide(d18:1/20:1 OH) (0.026) dAMP (0.027) PG(32:2) (0.032) PC(16:0/18:2) (0.033) PI(36:1) (0.034)	Hydroxyproline (< 0.001) 1-Pyrroline-5-carboxylic acid (< 0.001) PC(18:1/22:5) (< 0.001) PC(22:6/P-18:0) (< 0.001) PC(36:0) (0.006) PC(18:1/22:6) (0.008) Adipoylcarnitine (0.017) PC(16:0/22:6) (0.025) Arginine (0.030) PC(38:5) (0.031) Gluconic acid (0.032) Vitamin K2 (0.033) Glucosamine 6-phosphate (0.034)
	612	61-Males	PC(16:0/16:0) (< 0.001) GC(18:1/16:0) (< 0.001) Ceramide(d18:1/16:0) (< 0.001) THC 18:1/24:0 (< 0.001) Ceramide(d18:1/24:2) (< 0.001) PI(38:4) (< 0.001) DHC(18:1/16:0) (< 0.001) PA(16:0/16:0) (< 0.001) SM(d18:1/22:1 OH) (< 0.001) SM(d18:1/24:2 OH) (< 0.001) Ceramide(d18:1/16:1 OH) (< 0.001) SM(d18:1/22:0) (< 0.001) Ethanolamine (< 0.001) FAD (< 0.001) 4-Hydroxyphenyllactic (< 0.001) Ceramide(d18:1/16:1) (< 0.001) Ceramide(d18:1/18:0) (< 0.001) SM(d18:1/16:0) (< 0.001) SM(d18:1/18:2 OH) (< 0.001) Ceramide(d18:1/24:1) (< 0.001) Ceramide(d18:1/26:2) (< 0.001) Ceramide(d18:1/25:0) (0.005) Ceramide(d18:1/22:2) (0.005) Ceramide(d18:1/22:1) (0.005) Behenic acid (0.008) Hydroxyisocaproic acid (0.010) Uric acid (0.010) Pyroglutamic acid (0.012) SM(d18:1/24:0) (0.012) Lathosterol (0.011) PC(16:0/20:4) (0.011) SM(d18:1/22:1) (0.013) Ceramide(d18:1/22:0) (0.014) SM(d18:1/16:0 OH) (0.015) Ceramide(d18:1/24:0) (0.016) SM(d18:1/20:2 OH) (0.016) PC(18:1/18:1) (0.017) Ceramide(d18:1/16:2 OH) (0.018) PI(38:3) (0.021) 2-Methylcitric acid (0.021) SM(d18:1/22:0 OH) (0.022) 24,25-Epoxycholesterol (0.022) Cholesterol (0.023) SM(d18:1/18:0) (0.024) Ceramide(d18:1/24:0 OH) (0.026) SM(d18:1/22:2) (0.026) 2-Hydroxy-3-methylbutyrate (0.027) Ceramide(d18:1/16:0 OH) (0.028) Deoxyguanosine (0.028) Tiglylcarnitine (0.033) SM(d18:1/16:1 OH) (0.032) Ceramide(d18:1/18:1 OH) (0.031) SM(d18:1/25:0) (0.032)	1-Pyrroline-5-carboxylic acid (< 0.001) l-Serine (< 0.001) Arginine (< 0.001) Methionine sulfoxide (< 0.001) PC(18:1/22:6) (0.012) PC(20:5/P-16:0) (0.019) l-Threonine (0.021) Gamma-Aminobutyric acid (0.028)
Armstrong et al. (2015) [26]	29	6	Acetate (0.040) Glutamate (0.029) Hypoxanthine (0.001) Lactate (0.006) Phenylalaine (0.001)	Glucose (0.011)
Armstrong et al. (2012) [18]	22	2	Glutamine (0.002) Ornithine (0.045)	None
Jones et al. (2005) [27]	26	4	Taurine (< 0.001) Histidine (< 0.001) Tyrosine (< 0.01) Alpha-amino-*n*-butyric acid (< 0.05)	None

**Table 3:** Major biochemical pathways associated with blood metabolite differences in CFS/ME/SEID patients

Author and year	Major biochemical pathways
Germain et al. (2020) [21]	Energy metabolism Amino acid metabolism Lipids Fatty acid metabolism Androgenic steroids Corticosteroids Secondary bile acid metabolism Xenobiotics Chemical Food component/plant
Germain et al. (2018) [22]	Energy metabolism Tricarboxylic acid cycle Amino acid metabolism Nucleotides Pyrimidine metabolism Purine metabolism Peptides Protein degradation Cofactors and vitamins Haeme Vitamin E pathway
Nagy-Szakal et al. (2018) [29]	Energy metabolism Transport of activated residues between cellular organelles Lipid metabolism Cell membrane phospholipid
Germain et al. (2017) [23]	Energy metabolism ATP and ADP perturbations Amino acid metabolism Pentose phosphate pathway Ascorbate and aldarate metabolism Glycolysis pathway Gluconeogenesis pathway Citrate cycle Starch and sucrose metabolism Galactose metabolism Pyruvate metabolism Nucleotides Purine metabolism Lipids metabolism Biological membrane composition
Yamano et al. (2016) [24]	Energy metabolism Tricarboxylic acid cycle Urea cycle
Fluge et al. (2016) [25]	Energy metabolism Amino acid metabolism
Naviaux et al. (2016) [30]	Energy metabolism Redox regulation NADPH availability
Armstrong et al. (2015) [26]	Energy metabolism Glycolysis pathway amino acid metabolism
Armstrong et al. (2012) [18]	Energy metabolism Amino acid metabolism (urea pathway) Nitrogen metabolism (urea pathway)
Jones et al. (2005) [27]	Energy metabolism Amino acid metabolism

### Urine metabolites—secondary outcome

Three studies assessed a total of 84 metabolites in urine samples from CFS/ME/SEID patients and HC. A total of 19 metabolites were determined to be significantly different between the two groups (Table 4). One study reported an unidentified metabolite in CFS/ME/SEID patients and it was named Chronic Fatigue Syndrome Urinary Metabolite (CFSUM) [28]. However, since the paper was published in 1996, CFSUM has been identified to be partial derivatives of other metabolites and no further studies have reported this finding.

**Table 4:** Study results for urinary metabolite analyses

Author	Number of metabolites		Urinary metabolite	
	Assessed	Significantly different in CFS/ME/SEID vs HC	Decreased in CFS/ME/SEID vs HC (p-value)	Increased in CFS/ME/SEID vs HC (p-value)
Armstrong et al. [26]	30	5	Acetate (0.003) Alanine (0.049) Formate (0.002) Pyruvate (0.034) Serine (0.034)	None
Jones et al. [27]	26	6	Beta-alanine (< 0.05) Hydroxyproline (< 0.001) Histidine (< 0.05) Methionine (< 0.01) Cystine (< 0.01) Phenylalanine (< 0.01)	None
McGregor et al. [28]	28	8	CFSUM2 (< 0.001) Alanine (< 0.005) Glutamic acid (< 0.02)	Aminohydroxy-*N*-methylpyrrolidine (< 0.001) Tyrosine (0.02) Beta-Alanine (< 0.02) Succinic acid (< 0.05) Aconitic acid (< 0.05)

### Quality assessment

All studies included in this review were assessed for quality and bias by two authors (TKH and NEF) using the JBI Critical Appraisal Checklist for Case Control Studies (Additional file 1). Results of the two authors revealed no discrepancies with the quality assessment of each study included in this review. No studies were excluded due to low quality assessment scores. Across the included studies, the average score was 84 per cent (range: 57 to 100 per cent) and two of the papers scored 100 per cent [22, 29]. Outcomes were assessed in a standard, valid and reliable way with appropriate statistical analysis for comparisons between CFS/ME/SEID patients and HC in 100 per cent of studies. Identification criteria for CFS/ME/SEID patients and HC was used in 90 per cent of the included studies [18, 21, 22, 24–30]. One study did not detail any inclusion or exclusion criteria for the HC [23]. CFS/ME/SEID patients were most commonly matched by age and sex in 72 per cent of the studies [18, 21–23, 26–30]. Sufficient detail for how the participants were sourced was provided in 64 per cent of the studies [21–25, 29, 30]. Identification of confounding factors and strategies utilised to control for the confounding factors were addressed in 82 per cent of the studies [18, 21, 22, 24–29]. Exclusion criteria in the study design was the most common method used to control for confounding factors.

## Discussion

The purpose of this systematic review was to summarise the current literature to determine if there was any evidence to suggest that a dysregulated metabolomic profile may contribute to the pathogenesis of CFS/ME/SEID. Despite no studies being excluded from this review on the bases of a low-quality assessment score, a number of different methods were employed to measure and compare metabolites in CFS/ME/SEID patients to HC. Hence, this systematic review reports that a lack of consistency with scientific research design provides no evidence for metabolomics to be clearly defined as a contributing factor to the pathogenesis of CFS/ME/SEID.

### Study participants

Across the 11 studies included in this review, four different diagnostic criteria including 1994 Fukuda, CCC, Oxford and IOM were used to diagnose CFS/ME/SEID patients. The use of the different criteria in this review makes it inherently difficult to draw reliable conclusions from the results as each definition varies in the selection criteria for the symptoms, illness onset and duration of fatigue suffered [31]. The 1994 Fukuda definition was used in eight out of the ten studies. It has been argued that the 1994 Fukuda definition is limited by specificity due to its broad and non-specific criteria, which causes an inconsistent identification of CFS/ME/SEID cases for research purposes [31]. The IOM definition was used in one study as it was designed to allow for a broader clinical criteria to identify CFS/ME/SEID patients. However, the IOM is limited as it only has a few exclusionary conditions [32]. It has also been reported that when results for the IOM are compared to the 1994 Fukuda, the IOM criteria results in a higher prevalence rate and a classification system that is more heterogeneous [30, 32].

It has been recommended that the revised criteria including the CCC or the ICC be used for research purposes. These two definitions allow for a more consistent identification of CFS/ME/SEID patients as they employ a more stringent set of criteria and consider the multisystemic nature of symptoms experienced by patients. For example, the ICC included revision of CFS/ME/SEID symptoms into categories including (a) neurological impairments, (b) immune, gastro-intestinal or genito-urinary impairment and (c) energy metabolism or transport impairments [7]. In addition, the ICC allows for the categorisation of CFS/ME/SEID patients according to symptom severity [7]. As no studies in this review used the ICC, no data were collected on the symptom severity of CFS/ME/SEID patients. This is a potential recruitment bias as the other definitions favour CFS/ME/SEID patients who can self-present to clinics, hospitals and universities for sample collection, thus excluding bed- or house- bound CFS/ME/SEID patients from participating. This sampling bias is likely to confound results as it limits CFS/ME/SEID participant selection to a mild or moderate illness severity.

CFS/ME/SEID has a higher prevalence in females when compared to males, with the ratio reported to be as high as 6:1 [33]. Based on this finding, four out of the ten studies in this review recruited only female CFS/ME/SEID and HC participants [21–23, 26]. Two studies reported that the separation of the metabolite data sets into males and females revealed different significant results, leading to the conclusion that sex may influence the interpretation of the results and warranted consideration for future studies [25, 30]. Eight out of the ten studies age- and sex- matched CFS/ME/SEID patients with HC to minimise any variation [18, 22, 23, 26–30].

### Metabolite extraction and analysis

From the 11 studies included in this review, two studies used the same Nuclear Magnetic Resonance Spectrometry analysis method and two studies used the same Metabolon Mass Spectrometry method [18, 21, 22, 26]. The remainder of the studies used different metabolite extraction and analysis methods. Differences in methods included metabolite extraction using liquid–liquid procedures followed by Nuclear Magnetic Resonance spectrometry to detect the metabolites. Other methods used gas chromatography or capillary electrophoresis coupled to mass spectrometry to separate the metabolites prior to detection [18, 28, 29]. It was also identified that different reagents were used in the extraction process. Each of the extraction separation techniques and mass spectrometry methods detects differences in metabolites to varying degrees depending on the chemical properties [18]. This is a confounding factor that may contribute to the inconsistencies reported for metabolomics in CFS/ME/SEID patients. A lack of sampling standardisation may also contribute to the differences in results reported in the literature. In addition, both untargeted and targeted techniques were employed for metabolite detection in this review. However, given the limited information available on the potential role of metabolomics in the pathogenesis of CFS/ME/SEID, it has been recommended that the metabolomics studies should be untargeted [22]. This may allow for pattern identification that pending results, can be tailored down to a targeted analysis for future screenings. These findings on metabolite extraction and analysis techniques suggest that there is currently no standardised protocol for investing metabolomics.

### Metabolites

A total of 210 blood metabolites and 19 urine metabolites were found to be significantly different between CFS/ME/SEID patients and HC. Impairments in the energy metabolism pathway were identified in all of the included studies and amino acid metabolism was identified to be dysfunctional in seven of the studies. One proposed mechanism is that dysfunctional energy metabolism causes a hypometabolic state that may contribute to the fatigue experienced by CFS/ME/SEID patients [18, 24, 30]. However, when evaluating the specific metabolites associated with each major biochemical pathway in CFS/ME/SEID patients, inconsistent results were observed, which may be explained by a number of reasons. As described above, the discrepancies may be attributed to differences in the diagnostic criteria for CFS/ME/SEID patients and the metabolite extraction and analysis method. The blood metabolites were also measured in plasma and serum samples. Differences in metabolite levels have been identified between these sample types, where significantly more metabolites have been found in serum samples when compared to plasma samples [25, 34]. This is a confounding factor that makes it difficult to draw conclusions about the metabolite results in plasma and serum samples from CFS/ME/SEID patients.

A number of additional confounding factors have also been identified to influence metabolite levels, which may have contributed to discrepancies in this review. At any given point, the metabolic state of an individual is influenced by a number of factors including current conditions, age, body mass index, physical activity levels, timing and magnitude of exposures to emotional and physical stress, trauma, diet, nutritional and natural remedy supplements, infections and the microbiome [25, 30]. In particular, studies in this review identified that higher body mass index, illness duration, physical activity, dietary supplements, medications, clinical severity and fasting before blood collection were all correlated with metabolite results [25, 30]. Eight of the studies included in this review reported taking measures to control for the influence that diet, nutritional and natural remedy supplements and medication may have on metabolite levels. Furthermore, the dilution factor of blood and urine has also been identified as a confounding factor, highlighting the importance of using a standardisation tool when measuring total metabolite concentrations [18, 26]. These findings suggest that future research should include appropriate ways to control or minimise the influence of these confounding factors in the study design.

It is known that a number of metabolites can influence a diverse range of biological functions. This makes it difficult to determine if a significant metabolite result contributes to the pathogenesis and clinical presentation of CFS/ME/SEID. For example, deficiencies were identified in adenosine triphosphate, adenosine diphosphate, cyclic adenosine monophosphate and inosine monophosphate [22, 23]. These metabolites are critical for energy metabolism and they are also essential for the function of additional pathways including purine metabolism, intra-cellular signal transduction and chemical energy storage in muscles [22]. In this case, the multifunctional roles of a number of metabolites does not enable a link of specific metabolites to a single metabolic pathway in CFS/ME/SEID patients. This makes it difficult to draw conclusions about the metabolic and clinical significance of these findings. It has also been identified that metabolic dysregulations can present as the aftermath of complex interaction with genes, transcripts and proteins [25, 30]. This draws attention to the need to determine if dysregulated metabolomics in CFS/ME/SEID is a cause or consequence of the illness.

This review included metabolite measurements in both blood and urine samples as it is known that there is a shared relationship between the metabolites within these samples [18, 35]. The breakdown products of metabolic processes are excreted in urine, and it is proposed that if there is a disconnection between this process, it can indicate a disruption of homeostatic mechanisms [18, 36]. For example, changes in blood metabolites may indicate the presence of impaired metabolism, whilst changes observed in urine metabolites may suggest that there is a deficiency or excess in the blood that has been required to be excreted in order to maintain homeostasis [18]. One study included in this review examined metabolites in blood and urine samples from CFS/ME/SEID patients, however, no correlation analysis was completed between the samples for interpretation [27]. Furthermore, urinary excretion patterns of metabolites need to be interpreted with caution due to the role of kidney function in this process. Kidney function would need to be determined in all participants and standardised using creatinine clearance protocols.

### Quality assessment

The quality assessment was mostly consistent for the studies included in this review. Eight out of the 11 studies appropriately age- and sex-matched the CFS/ME/SEID patients to HC in order to minimise variation. Four studies were limited as they did not provide any information detailing how their participants were recruited. All studies used an internationally accepted diagnostic criteria to identify CFS/ME/SEID patients, however for reasons described above, the use of different criteria makes it difficult to draw consistent conclusions regarding metabolites in CFS/ME/SEID. One study did not report any criteria used for the selection of the HC. Given the influence that confounding factors have on the measurement of metabolites as described above, nine of the studies included in this review controlled for these factors through study design. All of the included studies used accepted laboratory methods to measure metabolites. Statistical analysis was also appropriately used in all studies to identify significant differences between CFS/ME/SEID patients and HC.

Quality assessments are usually measured on a spectrum and one limitation of the JBI quality assessment is that the checklist appraisal only allows a categorical assessment as yes, no or not applicable. This has the potential to allow for the introduction of inter-reviewer bias. In addition, a further limitation is that the JBI criteria does not recommend a threshold level for when studies should be included or excluded based on a scoring system graded as good, moderate or poor.

### Future research

Future well designed metabolomic CFS/ME/SEID research investigations are required to provide evidence for the pathogenesis of CFS/ME/SEID. Larger sample sizes of CFS/ME/SEID patients and HC would increase the power of the studies. All further studies should also use the same diagnostic criteria for CFS/ME/SEID, preferably the ICC due to its stringent criteria for identifying CFS/ME/SEID patients and for the categorisation of patients based on symptom severity. A consistent use of the same metabolite extraction and analysis method would also be beneficial to remove any influence of methodological variations. Strict controlling of the confounding variables described above through study design would also help to reduce any interactions that may interfere with metabolite measurement. In addition, comparison of CFS/ME/SEID metabolomics with other diseases where fatigue is also a core symptom would help to determine if any dysregulation is unique to CFS/ME/SEID.

## Conclusion

The aim of this systematic review was to evaluate if there is any evidence of metabolomics contributing to the pathogenesis of CFS/ME/SEID. The findings of this review suggest that methodological variations between the included studies make it difficult to draw consistent conclusions regarding the possibility of metabolomics contributing to the pathogenesis of CFS/ME/SEID. Further research using the same CFS/ME/SEID diagnostic criteria, metabolite analysis method and control of the confounding factors that influence metabolite levels is required. Based on the data currently available, metabolomics does not contribute to the pathogenesis of CFS/ME/SEID.

## Supplementary information

**Additional file 1: Table S1.** The Joanna Briggs Institute (JBI) Critical Appraisal Checklist for Case Control Studies. This file also contains the JBI Critical Appraisal justification for each of the included studies.

## Data Availability

All data generated or analysed during this study are included in this published article [and its additional information files].

## Abbreviations

CCC: Canadian Consensus Criteria
CFS/ME/SEID: Chronic Fatigue Syndrome/Myalgic Encephalomyelitis/Systemic Exertion Intolerance Disease
CFSUM: Chronic Fatigue Syndrome Urinary Metabolite
HC: Healthy controls
ICC: International Consensus Criteria
IOM: Institute of Medicine
JBI: Joanna Briggs Institute
MeSH: Medical subject headings
PRIMSA: Preferred Reporting Items for Systematic Reviews
