# Supplementary material for: A systematic review of metabolomic dysregulation in Chronic Fatigue Syndrome/Myalgic Encephalomyelitis/Systemic Exertion Intolerance Disease (CFS/ME/SEID)
Source: J Transl Med. 2020 May 13;18:198. doi: 10.1186/s12967-020-02356-2 (PMC7222338; doi:10.1186/s12967-020-02356-2)
Supplement: Supplementary file 1 — Additional file 1: Table S1. The Joanna Briggs Institute (JBI) Critical Appraisal Checklist for Case Control Studies. This file also contains the JBI Critical Appraisal justification for each of the included studies. [file 12967_2020_2356_MOESM1_ESM.docx]

**Additional File 1**

**Table S1:** The Joanna Briggs Institute (JBI) Critical Appraisal Checklist for Case Control Studies.

| **Author** | **Year** | **1** | **2** | **3** | **4** | **5** | **6** | **7** | **8** | **9** | **10** | **Score (%)** |
| --- | --- | --- | --- | --- | --- | --- | --- | --- | --- | --- | --- | --- |
| Germain *et al.* | 2020 | N | Y | Y | N/A | N/A | Y | Y | Y | N/A | Y | 86 |
| Germain *et al.* | 2018 | Y | Y | Y | N/A | N/A | Y | Y | Y | N/A | Y | 100 |
| Nagy-Szakal *et al.* | 2018 | Y | Y | Y | N/A | N/A | Y | Y | Y | N/A | Y | 100 |
| Germain *et al.* | 2017 | Y | Y | N | N/A | N/A | N | N | Y | N/A | Y | 57 |
| Yamano *et al.* | 2016 | N | Y | Y | N/A | N/A | Y | Y | Y | N/A | Y | 86 |
| Fluge *et al.* | 2016 | N | Y | Y | N/A | N/A | Y | Y | Y | N/A | Y | 86 |
| Naviaux *et al.* | 2016 | Y | Y | Y | N/A | N/A | N | N | Y | N/A | Y | 71 |
| Armstrong *et al.* | 2015 | Y | N | Y | N/A | N/A | Y | Y | Y | N/A | Y | 86 |
| Armstrong *et al.* | 2012 | Y | N | Y | N/A | N/A | Y | Y | Y | N/A | Y | 86 |
| Jones *et al.* | 2005 | Y | N | Y | N/A | N/A | Y | Y | Y | N/A | Y | 86 |
| McGregor *et al.* | 1996 | Y | N | Y | N/A | N/A | Y | Y | Y | N/A | Y | 86 |

Abbreviations: Y - Yes; N - No; U – unclear; N/A - not applicable. JBI Checklist items answered as N/A were removed from the final percentage score.

JBI Checklist items:

1. Were the groups comparable other than the presence of disease in cases or the absence of disease in controls?
2. Were cases and controls matched appropriately?
3. Were the same criteria used for identification of cases and controls?
4. Was exposure measured in a standard, valid and reliable way?
5. Was exposure measured in the same way for cases and controls?
6. Were confounding factors identified?
7. Were strategies to deal with confounding factors stated?
8. Were outcomes assessed in a standard, valid and reliable way for cases and controls?
9. Was the exposure period of interest long enough to be meaningful?
10. Was appropriate statistical analysis used?

**JBI Critical Appraisal Justification**

Germain *et al.* 2020

1. All female and body mass index- matched participants. Significant difference reported in ages between CFS/ME/SEID patients and HC.
2. CFS/ME/SEID and healthy control participants recruited from an existing cohort from Dr Susan Levine in Manhattan, NY.
3. All CFS/ME/SEID patients met 1994 Fukuda definition. Healthy controls met the major criteria of having no acute or chronic fatiguing illnesses.
4. No exposure.
5. No exposure.
6. Confounders identified in exclusion criteria for CFS/ME/SEID patients and healthy controls (no acute or chronic fatiguing illnesses).
7. Controlled for by exclusion in study design.
8. Untargeted metabolite measurement in plasma samples using Precision Metabolomics ultra-high-performance liquid chromatography /tandem accurate mass spectrometry at Metabolon, Morrisville, NC.
9. No exposure.
10. A non-parametric Wilcoxon rank-sum test was used to compared metabolite results between CFS/ME/SEID patients and HC (p < 0.05).

Germain *et al.* 2018

1. All female, age- and body mass index- matched participants.
2. CFS/ME/SEID and healthy control participants recruited from an existing cohort from Dr Susan Levine in Manhatten, NY.
3. All CFS/ME/SEID patients met 1994 Fukuda definition. Healthy controls met the major criteria of having no acute or chronic fatiguing illnesses.
4. No exposure.
5. No exposure.
6. Confounders identified in exclusion criteria for CFS/ME/SEID patients and healthy controls (no acute or chronic fatiguing illnesses).
7. Controlled for by exclusion in study design.
8. Untargeted metabolite measurement in plasma samples using Precision Metabolomics ultra-high-performance liquid chromatography /tandem accurate mass spectrometry at Metabolon, Morrisville, NC.
9. No exposure.
10. 356 metabolites failed the Shapiro test of normality (*p* < 0.05) and it was concluded that the non-parametric Wilcoxon rank-sum test (*p* < 0.05) was more appropriate than a *t*-test. In order to adjust for multiple comparison testing, the Benjamini-Hochberg (BH) correction was applied to the *p­*-values to control for false discovery rate.

Nagy-Szakal *et al.* 2018

1. All participants matched according to age-, sex-, race/ethnicity-, geographical/ clinical site and season of sampling. No differentiation of male and female proportion in cohorts without irritable bowel syndrome.
2. CFS/ME/SEID patients and healthy controls recruited from the Chronic Fatigue Initiative (CFI) cohort across four sites in the United States of America (New York, NY; Salt Lake City, UT; Incline Village, NV; and Miami, FL).
3. All CFS/ME/SEID patients met 1994 Fukuda definition and/or Canadian Consensus Criteria and completed standardised screening and assessment instruments including medical history and symptom rating scales, had a physical examination and provided blood samples. All healthy controls met the major criteria of having no self-reported CFS/ME/SEID symptoms or other conditions deemed by the recruiting physician to be non-representative of a healthy control population including: substance abuse in the prior year, any history of self-reported psychiatric illness, antibiotic use in the prior three months, immunomodulatory medications in the prior year and clinically significant findings on physical examination or screening laboratory tests. Supplementary data provides clinical and medical history assessments of all participants.
4. No exposure.
5. No exposure.
6. Confounders identified in exclusion criteria for healthy controls as described in item number 3. Exposome and vitamin metabolites (exogenous environmental compounds) excluded from biomarker analysis because vitamin supplements, medications and diet may impact metabolite levels independent of disease status. Exclusion of 5-methoxytryptamine from analysis as it is confounded by the use of anti-depressants in 50% of cases.
7. Controlled for by exclusion in study design.
8. Untargeted and targeted metabolite measurement in plasma samples using gas chromatography time-of-flight and liquid chromatography-tandem mass spectrometry instruments by the West Coast Metabolomics Centre at University of California.
9. No exposure.
10. The non-parametric Mann-Whitney U test (*p* < 0.05) and adjusted univariate logistic regression (*p* < 0.05) were applied to identify potential predictors differentiating CFS/ME/SEID from controls.

Germain *et al.* 2017

1. All female, age(within 2 years)- and body mass index- matched participants.
2. CFS/ME/SEID and healthy control participants recruited from a cohort of participants from Dr Susan Levine in Manhatten, NY.
3. All CFS/ME/SEID patients met 1994 Fukuda definition. No criteria listed for the selection or inclusion of the healthy controls.
4. No exposure.
5. No exposure.
6. No identification of confounding factors in the paper. Taurine stated to be taken as a nutritional supplement by CFS/ME/SEID patients and Taurine was identified to be significantly lower in CFS/ME/SEID patients.
7. No identification of measures taken to control for confounding factors.
8. Untargeted metabolite measurement in plasma samples using Q-Exactive Mass Spectrometry at Cornell University.
9. No exposure.
10. Testing for statistical difference between the data sets for CFS/ME/SEID patients and HC was completed using two statistical tests. For no assumption regarding the distribution of the data, the non-parametric Kruskal-Wallis test (*p* <0.05) was used. For the assumption of a normal distribution, the parametric Student’s *t*-test (*p* <0.05) after log10 transformation was used. In order to adjust for multiple comparison testing, the Benjamini-Hochberg (BH) correction (*Q* <0.15) was applied to the *p­*-values to control for false discovery rate.

Yamano *et al.* 2016

1. Does not describe how CFS/ME/SEID patients and healthy controls were matched.
2. CFS/ME/SEID patients were recruited from the Fatigue Clinical Centre of Osaka City University Hospital (Osaka, Japan). Healthy controls were recruited via online advertisements and shift workers were excluded.
3. All CFS/ME/SEID patients met 1994 Fukuda definition. Subjects with psychiatric disorders or with chronic diseases that are sometimes accompanied by fatigue (eg. cancer, diabetes) and those taking medications known to affect autonomic nerve function or the central nervous system were excluded.
4. No exposure.
5. No exposure.
6. Confounders identified in exclusion criteria for CFS/ME/SEID patients and healthy controls as described above in item number three.
7. Controlled for by exclusion in study design.
8. Untargeted metabolite measurement in plasma samples using Capillary electrophoresis time-of-flight mass spectrometry. Data sets provided for a training and validation analysis.
9. No exposure.
10. The non-parametric Mann-Whitney U test (*p* < 0.05) was used to assess the statistical significance of differences between CFS/ME/SEID patients and healthy controls. Demographic data were compared between the groups using Student’s *t*-test (*p* < 0.05).

Fluge *et al.* 2016

1. Does not describe how CFS/ME/SEID patients and healthy controls were matched.
2. CFS/ME/SEID patients were participants in three separate clinical trials in Norway and had blood samples collected before intervention (baseline). The healthy controls were recrutied from blood donors at Haukeland University Hospital and staff at the Department of Oncology, Haukeland University Hospital.
3. All CFS/ME/SEID patients met the Canadian Consensus Criteria. The healthy controls had no chronic disease and no chronic medication used.
4. No exposure.
5. No exposure.
6. Of the 200 CFS/ME/SEID patients, 47 patients were identified to have fasted overnight, a confounding factor when compared to the 153 CFS/ME/SEID patients and 102 healthy controls who did not fast. The concentration of the amino acid alanine is known to be highly influenced by the presence of metabolites in other metabolic pathways, which was identified as a confounding factor. The paper also identification that other clinical variables (eg. age, BMI, level of physical activity, CFS/ME/SEID severity and CFS/ME/SEID disease duration) may have a confounding effect on the results of the amino acid levels measured in serum.
7. Separation of the overnight fasting CFS/ME/SEID patients in data analyses to avoid a confounding effect. The serum concentration of alanine was analysed separately to negate any confounding effect on the results. To evaluate the potential influence of confounding clinical variables (listed in item number six above) on the observed changes in amino acids measured in the serum, an ANOVA correlation analysis was performed.
8. Targeted metabolite measurement in serum samples using gas chromatography-tandem mass spectrometry.
9. No exposure.
10. Statistical analysis performed using SPSS version 23 and Graphpad prism version 7.0. Unpaired 2-tailed Student’s *t*-test (*p* <0.05) was used to compare the mean serum levels of amino acids between CFS/ME/SEID patients and healthy controls.

Naviaux *et al.* 2016

1. All participants matched according to age- and sex.
2. The 84 participants in the study were recruited from 51 zip codes around the United States and Canada.
3. All CFS/ME/SEID patients met the Fukuda, Canadian and Institute of Medicine diagnostic criteria. Healthy controls were participants who were age- and sex- matched volunteers without CFS/ME/SEID.
4. No exposure.
5. No exposure.
6. No identification or consideration to control for confounding factors in the paper.
7. No identification of measures taken to control for confounding factors.
8. Targeted metabolite measurement in plasma samples using hydrophilic interaction liquid chromatography, electrospray ionization, and tandem mass spectrometry in a single-injection method (Triple Quadrupole Mass Spectrometry).
9. No exposure.
10. The metabolomic data sets were log-transformed, scaled by control standard deviations and analysed by multivariate partial least squares-discriminant analysis, principal component analysis, Student’s *t*-test (*p* < 0.05), univariate ANOVA with pairwise comparisons. Post hoc correction for multiple hypothesis testing was completed using Fischer’s least significant difference method in MetaboAnalyst or the false discovery rate method of Benjamini and Hochberg.

Armstrong *et al.* 2015

1. All participants matched according to age- and sex.
2. Paper does not state how the CFS/ME/SEID patients or healthy controls were recruited.
3. All CFS/ME/SEID patients met the Canadian criteria. Diagnosis of CFS/ME/SEID was made on the nature of the clinical history, in association with the Canadian criteria, having excluded other illnesses. The same clinician also verified that subjects within the non-CFS/ME/SEID cohort were not suffering from CFS/ME/SEID or any other illnesses. All subjects were asked to list their current medications and oral supplements. None of the subjects were related to one another nor were they ever living together and none of the subjects were obese or nicotine dependent.
4. No exposure.
5. No exposure.
6. Identification of the influence of the dilution factor on absolute measurements of urine metabolites. Confounders identified in selection criteria for CFS/ME/SEID patients and healthy controls.
7. In order to remove the influence of dilution factor on both urine and blood metabolite measurements, each sample was normalised to the total metabolite concentration, producing relative abundance data. This method of normalisation focuses analysis on the ratio of metabolites within either the blood or urine. The confounding factors identified in participant selection were controlled for by exclusion in study design.
8. Untargeted metabolite measurement in serum and urine samples using Nuclear Magnetic Resonance Spectrometry.
9. No exposure.
10. Statistical analysis of the collected data comparing CFS/ME/SEID patients and healthy controls included Student’s *t -*test (*p* < 0.05), principal components analysis and Pearson correlations.

Armstrong *et al.* 2012

1. All participants age- and sex- matched.
2. Paper does not state how the CFS/ME/SEID patients or healthy controls were recruited.
3. All CFS/ME/SEID patients were diagnosed according to the Canadian criteria. None of the control subjects reported symptoms of fatigue. All subjects were asked to list any medications and oral supplements that they were taking at the time. None of the subjects were related to one another nor were they every living together. Two patients were removed from the dataset upon analysis due to large concentrations of glucose found in their blood consistent with an insulin-related disorder.
4. No exposure.
5. No exposure.
6. Confounders identified in selection criteria for CFS/ME/SEID patients and healthy controls.
7. The confounding factors identified in participant selection were controlled for by exclusion in study design.
8. Untargeted metabolite measurement in serum samples using Nuclear Magnetic Resonance Spectrometry.
9. No exposure.
10. All data was assessed for normality. The data not found to be normally distributed were either log converted in the raw data set or arcsine converted for the relatively distributed data set. Statistical analysis included Student’s *t -*test (*p* < 0.05) and Pearson correlation coefficients. Bootstrapping was employed to test the stability of the significant results from the Student’s *t -*test and Pearson correlations.

Jones *et al.* 2005

1. All participants age- and sex- matched.
2. Paper does not state how the CFS/ME/SEID patients or healthy controls were recruited.
3. All CFS/ME/SEID patients were diagnosed according to the Fukuda and Oxford criteria. Healthy controls were age- and sex-matched to CFS/ME/SEID patients. Subjects on medications and who were smokers were excluded. All CFS/ME/SEID patients and healthy controls completed a questionnaire (a shortened form of that of Ray et al.) to assess their somatic symptoms, cognitive difficulties, disability and recent course an any illnesses.
4. No exposure.
5. No exposure.
6. Confounders identified in selection criteria for CFS/ME/SEID patients and healthy controls.
7. The confounding factors identified in participant selection were controlled for by exclusion in study design.
8. Targeted metabolite measurement in plasma and urine samples using Reversed Phase Chromatography.
9. No exposure.
10. All data was tested for distribution normality and depending on the results, significant differences between CFS/ME/SEID patients and healthy controls were tested using unpaired independent Student’s *t -*test (*p* < 0.05) and Mann-Whitney *U* tests (*p* < 0.05).

McGregor *et al.* 1996

1. All participants age- and sex- matched.
2. Paper does not state how the CFS/ME/SEID patients were recruited. The healthy controls were recruited from the relatives of CFS/ME/SEID patients and from unrelated subjects.
3. All CFS/ME/SEID patients were diagnosed according to the Fukuda and Oxford criteria. Healthy controls were age- and sex- matched to CFS/ME/SEID patients. None of the control subjects reported fatigue that affected their lives. All subjects were asked to list the drugs and naturopathic remedies taken and the dietary changes that they had made within the preceding four weeks.
4. No exposure.
5. No exposure.
6. Confounders identified in selection criteria for CFS/ME/SEID patients and healthy controls.
7. The confounding factors identified in participant selection were controlled for by exclusion in study design. Two CFS/ME/SEID patients were excluded due to a medication taken in the seven days prior to specimen collection and the other was excluded as the urine specimen had been inappropriately collected. All urine samples were screened for drug related metabolites and none were detected.
8. Untargeted metabolite measurement in urine samples using capillary gas chromatography mass spectrometry.
9. No exposure.
10. All urine metabolite data (arcsine transformed) was analysed using Student’s *t -*test (*p* < 0.05).
